# Identification and Expression Profiling of Peripheral Olfactory Genes in the Parasitoid Wasp *Aphidius ervi* (Hymenoptera: Braconidae) Reared on Different Aphid Hosts

**DOI:** 10.3390/insects10110397

**Published:** 2019-11-08

**Authors:** Gabriel I. Ballesteros, Daniela A. Sepúlveda, Christian C. Figueroa

**Affiliations:** 1 Instituto de Ciencias Biológicas, Universidad de Talca, campus Talca 3460000, Chile; gballesteros@utalca.cl; 2 Centre for Molecular and Functional Ecology in Agroecosystems, Universidad de Talca, campus Talca 3460000, Chile; dani.sepulveda.14@gmail.com; 3 Facultad de Ciencias Agrarias, Universidad de Talca, campus Talca 3460000, Chile

**Keywords:** host fidelity, peripheral olfactory genes, olfaction, parasitoid wasps, inbreeding, biological control

## Abstract

Generalist parasitoids of aphids, such as the wasp *Aphidius ervi*, display significant differences in terms of host preference and host acceptance, depending on the host on which they developed (natal host), which is preferred over a non-natal host, a trait known as host fidelity. This trait allows females to quickly find hosts in heterogeneous environments, a process mediated by chemosensory/olfactory mechanisms, as parasitoids rely on olfaction and chemical cues during host selection. Thus, it is expected that proteins participating in chemosensory recognition, such as odorant-binding proteins (OBPs) and odorant receptors (ORs) would play a key role in host preference. In this study, we addressed the effect of parasitoid reciprocal host switching between two aphid hosts (*Sitobion avenae* and *Acyrthosiphon pisum*) on the expression patterns of chemosensory genes in the wasp *A. ervi.* First, by using a transcriptomic approach based on RNAseq of *A. ervi* females reared on *S. avenae* and *A. pisum*, we were able to annotate a total of 91 transcripts related to chemoperception. We also performed an in-silico expression analysis and found three OBPs and five ORs displaying different expression levels. Then, by using qRT-PCR amplification, we found significant differences in the expression levels of these eight genes when the parasitoids were reciprocally transplanted from *S. avenae* onto *A. pisum* and vice versa. This suggests that the expression levels of genes coding for odorant receptors and odorant-binding proteins would be regulated by the specific plant–aphid host complex where the parasitoids develop (maternal previous experience) and that chemosensory genes coding for olfactory mechanisms would play a crucial role on host preference and host acceptance, ultimately leading to the establishment of host fidelity in *A. ervi* parasitoids.

## 1. Introduction

Parasitoid wasps are a diverse group of hymenopterans that are natural enemies of a broad range of arthropods, including those of agronomic significance [1]. Adult parasitoids are free-living insects that can lay their eggs onto (exoparasitoids) or into (endoparasitoids) a host, which is subsequently killed during the larval development of the parasitoids [1]. Thus, parasitoids have an important role in the regulation of arthropod population sizes in natural environments [1] and have been used as biocontrol agents to reduce the population densities of target pest species [2,3]. This is the case of the endoparasitoid wasp *Aphidius ervi* Haliday (Hymenoptera: Braconidae)*,* a worldwide distributed koinobiont parasitoid of several aphid species [4]. Extensively used in biological control programs, *A. ervi* mainly parasitizes the pea aphid *Acyrthosiphon pisum* Harris (Hemiptera: Aphididae) [5,6], although it has become an important biocontrol agent of the grain aphid *Sitobion avenae* Fabricius (Hemiptera: Aphididae) [7,8]. However, the successful use of *A. ervi* in biological control programs depends on the specificity of host selection and host acceptance behaviors and on host suitability, as not all host species are suitable for parasitoids [9]. Thus, the reproductive success of *A. ervi* is intimately related to behavioral and foraging strategies used during the selection and parasitization of a suitable host [10].

Interestingly, maternal previous experience plays a key role in determining the host choice in the offspring, causing transgenerational phenotypic plasticity or maternal effect [11]. The oviposition preference in the offspring may follow the same host–plant system from which the parasitoid emerged (natal host), which has important ecological and evolutionary implications [5,12]. This phenomenon is known as host fidelity and is considered an important trait for the successful reproduction and progeny survival of parasitoid wasps [13]. Host fidelity should improve and maximize the reproductive performance of parasitoids on a target host [11,14], even in the case of naïve females without any previous experience [15]. Thus, parasitoid females must quickly locate and recognize a suitable host or habitat for oviposition, usually in chemically complex environments [16].

Host finding relies on the olfaction of environmental signals, such as specific chemicals and volatiles, emitted either from plants or from a host–plant complex, which are used by female wasps to carefully choose an appropriate host for oviposition [1,17,18]. However, neither background odors nor specific host-finding cues are fixed in nature [19]. Thus, the parasitoids would presumably modulate their olfactory system to encompass the composition of novel odorants and kairomones in response to variations in biotic (e.g., plant or host phenotype and genotype) and abiotic factors (e.g., wind speed, temperature, humidity) that would modify environmental odor profiles (i.e., scent environment; [20,21].

In adult parasitoids, it has been proposed that host preference would be a consequence of exposure, during larval stages, to both host- and host–plant-related chemical volatiles and cues (volatile organic compounds, VOCs) which are emitted by the plant–host complex upon aphid infestation [17] and which trigger behavioral responses upon recognition [22]. Hence, detection and processing of chemical signals play a crucial role during host searching and the selection process in adult parasitoids [23,24] and may be modulated by maternal experience and/or previous oviposition experience.

Given that olfactory behavioral responses depend on specific sets of proteins for odorant recognition and signal propagation and processing, it has been proposed that there is a molecular base underpinning the phenotypic plasticity of behavioral responses displayed by insects towards olfactory signals and cues [25]. Thus, variations in the ability to perceive and respond to chemosensory cues from the host or host–plant complex would also provide a target for adaptive evolution [26]. Indeed, phenotypic plasticity in the expression levels of chemosensory genes has been documented in response to different developmental, physiological, and social conditions [27] or even between individuals of the same species but exhibiting differences in their ecological preferences [28]. Thus, variation in the detection and processing of chemical signals is thought to be one of the main mechanisms driving the rapid responses of insects to varying environments and would be under transcriptional control rather than depending on sequence changes in coding sequences and proteins [29]. Hence, if the perception of chemical cues is modulated by the maternal previous experience, then it is crucial to disentangle its molecular base. This should be considered in attempts aiming to improve parasitoids’ efficacy as biological controller agents, as many parasitoid wasps are reared under laboratory conditions before they are released in the field [5,30].

However, as the aphid species parasitized by *A. ervi* differ in several biological aspects (e.g., host plant, host range, body size and color, composition of cuticular semiochemicals, cornicular secretions, defensive behaviors, etc.) [7], it is not clear if, in this species, the exposure to a novel aphid host (i.e., a non-natal plant–host complex) has an impact in terms of phenotypic plasticity on the expression levels of chemosensory genes. One way to test this is by comparing the expression levels of chemosensory genes of different *A. ervi* lineages reared on natal and non-natal hosts. If *A. ervi* uses the same strategy (in terms of olfactory/chemical recognition) for parasitizing both hosts, then the expression of chemosensory genes should be very similar. Alternatively, if *A. ervi* is able to modulate its olfactory system to encompass the composition of novel odorants and kairomones, then differences in terms of expression of chemosensory genes should be detected.

In this study, we addressed the effect of reciprocal host switching between aphid host species on the expression patterns of chemosensory genes on two *A. ervi* populations that naturally parasitize different aphid species (*A. pisum* and *S. avenae*). First, we used a transcriptomic approach based on RNAseq, to identify putative transcripts related to chemoperception in *A. ervi*. This approach allowed us to annotate chemosensory genes and characterize their expression levels when the parasitoids were reared on two different aphid hosts. Then, we studied if the exposure to a non-natal plant–host complex (regardless of the parasitoid lineage) had an impact on the expression of genes coding for odorant receptors (ORs) and odorant-binding proteins (OPBs, chemosensory genes). Thus, we compared the effects of host change from the natal aphid host to an alternative non-natal host on specific chemosensory genes, in order to outline the molecular mechanism underlying host fidelity establishment. Finally, as parasitoid wasps are usually reared in caged conditions before being released for biological control programs, which has been shown to increase inbreeding and reduce host fidelity [31], we compared the chemosensory gene expression profiles of *A. ervi* parasitoids sampled from field (exogamic) and laboratory (endogamic) populations.

## 2. Materials and Methods

### 2.1. Parasitoid Collection and Rearing

Parasitized individuals of *A. pisum* and *S. avenae* were collected as aphid mummies from fields of alfalfa (*Medicago sativa* L.) and wheat (*Triticum aestivum* L.), respectively, in Region del Maule, Chile (S 35°24′, W 71°40′). Aphid mummies were individually isolated in Petri dishes until adult parasitoid emergence. The emerged naïve parasitoids were then identified as *A. ervi* and sexed following standard taxonomic keys [32] under an Optika ST-155 (10×) binocular microscope. Stock laboratory lines of *A. ervi* parasitoids were founded from five naïve *A. ervi* virgin females and one naïve virgin male selected at random and obtained from the same aphid host population from which they were collected (*A. pisum* or *S. avenae*). Female and male individuals were left to mate in a Petri dish for 24 h with diluted honey and water for sustenance. Mated females were then transferred to a cage containing aphids ad libitum from the same species from which they emerged, with diluted honey and water for sustenance. The establishment of *A. ervi* parasitoids on their natal host for one single generation has been shown to erase any previous field experience (see [13]). Thus, two different stock lines of *A. ervi* populations were established in the laboratory (20 °C, D16/N8 photoperiod): (i) one *A. ervi* (Ae) population from *A. pisum* (AP, alfalfa race) maintained on broad bean (*Vicia faba* L.) (Ae–AP; natal host AP) and (ii) one *A. ervi* population from *S. avenae* (SA) maintained on barley (*Hordeum vulgare* L.) (Ae–SA; natal host SA). These aphids and their host plants have been used successfully for *A. ervi* rearing in previous studies [30,31,33]. All aphids used in this study were free of facultative endosymbiont bacteria, well-known to naturally occur in aphid populations [34,35], including the defensive endosymbiont *Hamiltonella defensa*, which confers protection against parasitoids [36,37].

### 2.2. Aphidius ervi RNASeq, Transcriptome Assembly, and Annotation

In this study, we used the *A. ervi* reference transcriptome (available at 10.6084/m9.figshare.4816939). Briefly, RNA was extracted from dissected heads and bodies of 60 female *A. ervi* parasitoids, which were collected alive from three caged parasitoid populations (*A. pisum*–Pea; *A. pisum*–Alfalfa and *S. avenae*–Barley; N = 20 per cage). For further details of the experiment, see reference [33]. After collection, total RNA was extracted using the RNEasy Plant Mini Kit (QIAGEN), and ribosomal RNA was depleted from total RNA using the Ribo-Zero rRNA Removal Kit for enrichment of both insect mRNA and non-poly-adenylated mRNA that might be present in *A. ervi* sequenced samples. The remaining RNA was used for library construction using the TruSeq Stranded Total RNA Sample Preparation Kit (Illumina) and sequenced using an Illumina HiSeq 2000 (2 × 100 bp, Paired End libraries; Macrogen, Korea). The raw RNA-seq libraries used in this study are available in NCBI (SRA database, accession PRJNA377544). Raw reads were assembled into a reference *A. ervi* transcriptome using Trinity 2.0.6 and annotated with BLASTx using the NR database (April 2016). Further details on both assembly and annotation are published elsewhere [33].

### 2.3. Annotation of Chemosensory Genes and Differential Expression Analysis

On the basis of the published *A. ervi* transcriptomic annotation table (available at https://doi.org/10.6084/m9.figshare.4822069.v1; [33]), we performed a search for putative chemosensory genes using keywords such as odorant receptor, odorant-binding protein, chemosensory protein, among others. Then, we analyzed the gene expression results for all putative chemosensory genes annotated in this transcriptome (Table 1) [33]. Briefly, gene expression was estimated by mapping RNA-seq libraries using Bowtie2 (ver. 2.2.4; [38]) and counting the mapped reads with the RSEM package [39]. Then, a count matrix was used as input for differential expression (DE) analysis, which was performed with the edgeR Bioconductor package. To allow inference when many tests are being conducted, the false discovery rate (FDR) was computed, which is the proportion of discoveries that are false among all discoveries [40]. Hence, genes that had at least 4-fold-changed values with an FDR-corrected *p* value of 0.01 or lower were considered as significantly differentially expressed between libraries/tissues (Table 1). All chemosensory genes displaying significant differences in their expression levels in the heads of *A. ervi* parasitoids reared on different aphid host–plant complexes (*S. avenae* and *A. pisum*; [33]) were considered as candidate genes and selected for further expression analysis using qRT-PCR (Table 2). Gene annotation was manually verified using BLASTx ver 2.7.0 against NCBI NR database (September 2017) for homology analysis with genes from other insect species such as *Drosophila melanogaster* M. (Diptera: Drosophilidae), which have been functionally characterized and for which odor response data are available (Table 3).

### 2.4. Reciprocal Transplant Experiments

To determine the effects of the rearing host on the expression of selected chemosensory genes in *A. ervi*, a reciprocal transplant experiment was conducted (Supplementary Figure S1), where the natal host corresponded to the control condition, and the non-natal host (i.e., the aphid host on which the parasitoids were transplanted) corresponded to the treatment. Aphid mummies from the first generation of each condition were isolated in Petri dishes until parasitoid emergence, and female parasitoids were mated with males (N = 30). Then, the mated females were randomly transplanted to rearing cages containing aphids ad libitum of the natal or non-natal hosts (reciprocal transplant) for two generations.

Aphid mummies from the third generation of each condition (natal and non-natal hosts) were isolated in Petri dishes until parasitoid emergence. Since mated females display a higher attraction to oviposition-site cues, virgin adult female parasitoids were left to mate with a male from the same condition for 24 h [46]. Each mated female was then transferred to an experimental arena (a modified 2 cm-diameter Petri dish) containing one single wingless aphid and a small piece of leaf from the plant where the aphid was feeding (i.e., broad bean for *A. pisum* and barley for *S. avenae*) [31]. After successful oviposition, each female was immediately stored in separate 1.5 mL microcentrifuge tubes containing RNALater (QIAGEN, Hilden, Germany) at −20 °C until dissection and RNA extraction. Previous studies have shown this procedure as suitable for addressing the formation of host fidelity in *A. ervi* wasps [30,31].

To determine the effects of long-time caged rearing (i.e., inbreeding) on the expression of chemosensory genes, we compared field populations of *A. ervi* parasitoids acclimated on natal or non-natal hosts for two generations (exogamic population) with *A. ervi* parasitoids from inbred populations that had been maintained in the laboratory on the same natal plant–host complex for more than 75 generations (endogamic population) (Supplementary Figure S1). The experimental individuals sampled from the inbred population corresponded to the same parasitoids studied in reference [31], which were preserved appropriately as described above.

### 2.5. RNA Extraction and cDNA Synthesis

We decided to study heads, as they contain most of the organs involved in chemosensory function and feeding, as well as most of the olfactory-associated proteins [47,48]. Female heads were dissected on ice using a sterile scalpel and pooled in a 1.5 mL microcentrifuge tube (N = 5 per pool). Total RNA was extracted using the RNEasy Plant Mini Kit (QIAGEN, Hilden, Germany) and eluted in 50 μL of RNAse-free water. The integrity of the RNA samples was assessed using a 1.1% gel by denaturing formaldehyde agarose gel electrophoresis, and the concentrations were estimated by spectrophotometry at 260 nm (Epoch Microplate Spectrophotometer, Biotek), resulting in the range of 4.26–8.17 ng/μL of total RNA for all samples. DNA traces were removed from the samples by DNase treatment using Turbo DNase (Ambion). Single-stranded cDNAs were synthesized using the SuperScript III Reverse Transcriptase System (Invitrogen). All procedures were conducted following the manufacturer’s instructions.

### 2.6. qRT-PCR Expression Analysis of OBPs and ORs

Determinations of the relative transcript abundance of eight chemosensation-related genes (five coding for ORs and three coding for OBPs, Table 3) were carried out by real-time PCR (qPCR) using cDNAs obtained from heads of *A. ervi* females transplanted to their natal or non-natal hosts (Supplementary Figure S1). For each selected target gene, specific primer pairs (listed in Table 2) were designed with Beacon Designer 8 software (Premier Biosoft) using the recently published *A. ervi* transcriptome to retrieve template sequences [33]. Each PCR reaction contained 2 μL of diluted cDNA (2 ng; 1 ng/μL), 10 μL Maxima SYBR Green PCR Master Mix (ThermoFisher Scientific), 6.4 μL of nuclease-free water, and 0.8 μL of each specific primer (1.6 μL for both forward and reverse primers; 10 mM concentration). Negative controls (nuclease-free water) were included for detecting any cross-contamination; positive controls for qPCR reactions were also included (*A. ervi* genomic DNA). All PCR reactions were carried out in triplicate (three technical replicates) using the Mx3000 P qPCR system (Stratagene, La Jolla, CA, USA) under the following cycling conditions: 95 °C for 10 min, 40 cycles of 95 °C for 30 s, 56 °C for 45 s, and 72 °C for 40 s. A dissociation curve was included immediately after each qPCR, using a ramp of 55–95 °C to confirm the absence of non-specific amplifications. All amplicons were sequenced to confirm the specific amplification of the target genes.

Expression data for each target gene were normalized using published primers which amplify Ribosomal Protein L19 of *A. ervi* [49] (primers listed in Table 2). Data from all *A. ervi* populations and rearing conditions were analyzed manually, and the relative transcript levels for each target gene were calculated using the comparative 2^−ΔΔCT^ method [50]. Each PCR reaction was performed in triplicate, and the mean of three biological replicates was calculated. Data were analyzed statistically by two-way ANOVA using GraphPad Prism version 6.01 (*p* value < 0.05). The expression of a given gene was compared between parasitoids reared on natal and non-natal hosts, considering the natal condition as the control. In the case of gene expression comparison between outbred and inbred *A. ervi* populations, the outbred condition was considered as the control.

## 3. Results

### 3.1. Identification of Putative Chemosensory Genes in the Reference A. ervi Transcriptome and in Silico Analysis of Expression Levels

We performed a thorough annotation for genes encoding OBPs, chemosensory proteins (CSPs), and ORs found within the *A. ervi* transcriptome [33]. We annotated 91 contigs belonging to gene families involved in insect chemoperception, including odorant binding proteins (OBPs; 10 transcripts), chemosensory proteins (CSPs; 2 transcripts), sensory neuron membrane proteins (SNMPs; 1 transcript), odorant receptors (ORs; 76 transcripts, including the conserved odorant co-receptor, ORco), and ionotropic receptors (IRs; 2 transcripts) [33]. Furthermore, using the same RNA-seq libraries, we detected several genes involved in chemical perception which displayed differential expression between *A. ervi* populations parasitizing different aphid hosts (Table 1).

### 3.2. qPCR Expression Levels of OBPs and ORs Genes in Parasitoids Reared on Natal and Non-Natal Hosts

**Figure 1:**
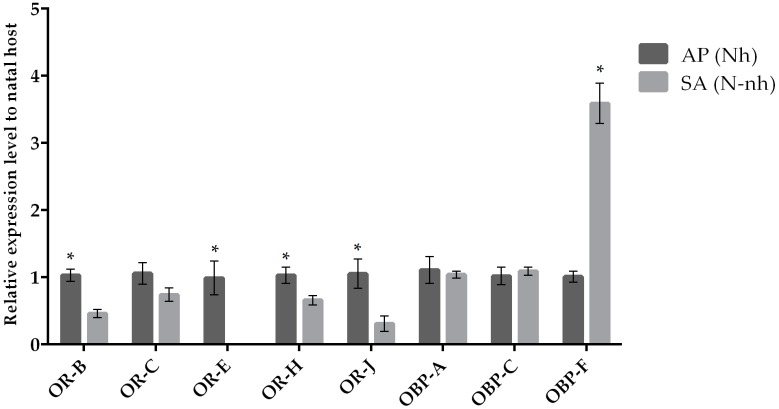
Mean (+/− SE) mRNA expression levels of ORs and OBPs from the heads of *A. ervi* maintained on the natal host *A. pisum* (AP; Nh) or on the non-natal host *S. avenae* (SA; N-nh) measured by RT-qPCR. RT-qPCRs were performed using specific primers for each gene. Normalizer gene: RPL19. The asterisk * above the bars indicates significant differences according to two-way ANOVA (*p* value < 0.05).

**Figure 2:**
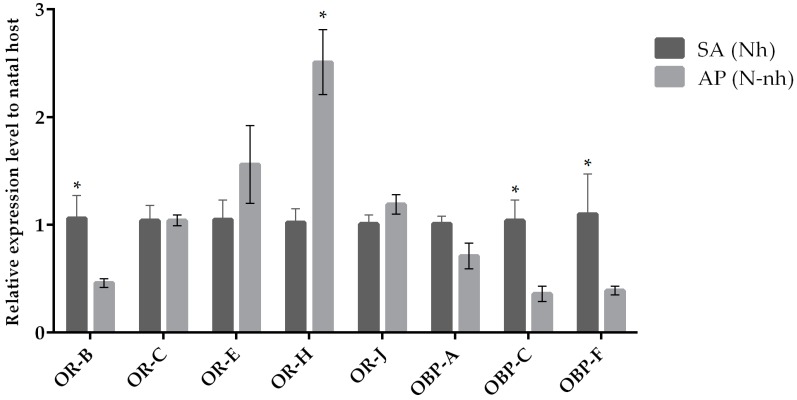
Mean (+/− SE) expression levels of ORs and OBPs from the heads of *A. ervi* maintained on the natal host SA (*S. avenae*; Nh) or on the non-natal host AP (*A. pisum*; N-nh) measured by RT-qPCR. RT-qPCRs were performed using specific primers for each gene. Normalizer gene: RPL19. The asterisk * above the bars indicates significant differences according to two-way ANOVA (*p* value < 0.05).

The expression of target chemosensation-related genes was assessed by qRT-PCR when parasitoid females from the same natal host were reared on their natal (control condition) and non-natal (experimental condition) hosts. We observed a variation in the expression levels when comparing chemosensory genes of parasitoids originated from *A. pisum* and *S. avenae* that were reared on their natal host or transplanted to non-natal hosts (Figure 1 and Figure 2). Our results indicate that the *OR-H* and *OBP-F* genes were up-regulated when *A. ervi* was reared on AP compared to SA (Figure 1 and Figure 2), regardless the natal host. Hence, rearing on AP increased the abundance of transcripts for both *OR-H* and *OBP-F* genes compared to rearing on SA. In contrast, the *OR-B* gene showed a reduced expression when the parasitoids were switched from their natal to non-natal hosts. A downregulation was detected for *OR-J* and *OR-E* when *A. ervi* was switched from AP (natal host) to SA (non-natal host), although no differences were observed for the reciprocal switch (SA to AP). Finally, *OBP-C* was downregulated when *A. ervi* was switched from SA to AP but not when the parasitoids were transplanted from AP to SA.

### 3.3. qPCR Expression Levels of OBPs and ORs in Parasitoids Reared on Different Natal Hosts but Transplanted on the Same Aphid Host

The expression of target chemosensation-related genes was assessed by qRT-PCR when parasitoid females from different natal hosts (AP and SA) were reared on non-natal aphid host species (Supplementary Figure S1). These comparisons aimed to determine whether rearing on the same aphid host species may alter the expression of chemosensory genes.

**Figure 3:**
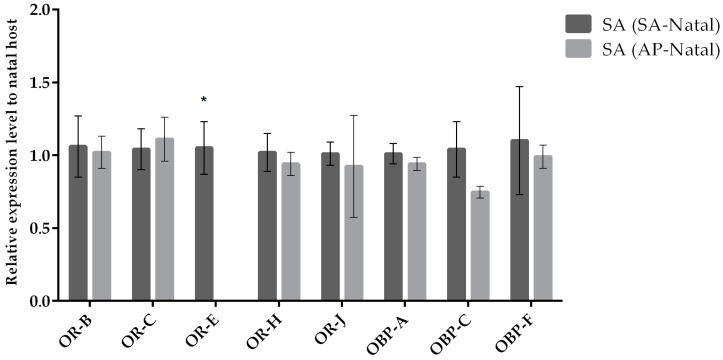
Mean (+/− SE) mRNA expression levels of ORs and OBPs from the heads of *A. ervi* maintained on the natal host *S. avenae* (SA-Natal) or switched from *A. pisum* to the non-natal host *S. avenae* (AP-Natal) measured by RT-qPCR. RT-qPCRs were performed using specific primers for each gene. Normalizer gene: RPL19. The asterisk * above the bars indicates significant differences according to two-way ANOVA (*p* value < 0.05).

**Figure 4:**
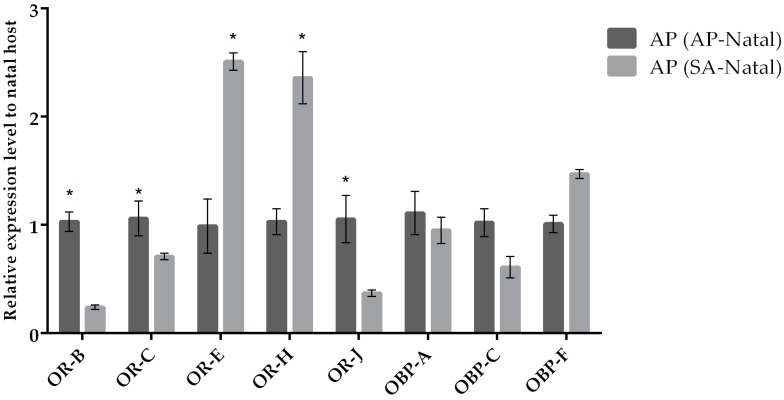
Mean (+/− SE) mRNA expression levels of ORs and OBPs from the heads of *A. ervi* maintained on the natal host *A. pisum* (AP-Natal) or switched from *S. avenae* to the non-natal host *A. pisum* (SA-Natal) measured by RT-qPCR. RT-qPCRs were performed using specific primers for each gene. Normalizer gene: RPL19. The asterisk * above the bars indicates significant differences according to two-way ANOVA (*p* value < 0.05).

**Figure 5:**
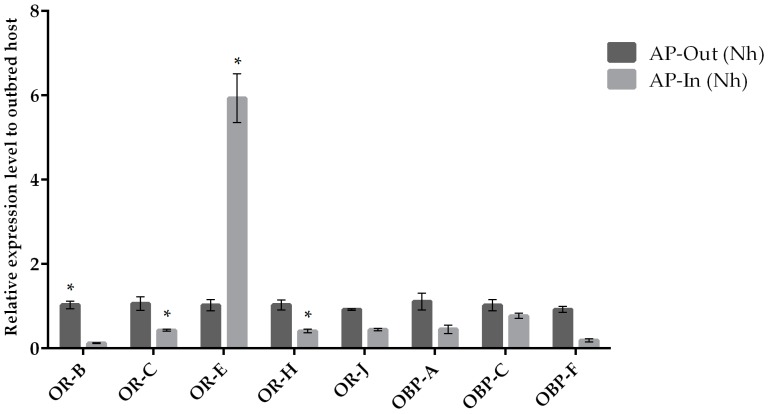
Mean (+/− SE) expression levels of ORs and OBPs from the heads of outbred (AP-exogamic) and inbred (AP-endogamic) *A. ervi* maintained on their natal host *A. pisum* (AP), measured by RT-qPCR. RT-qPCRs were performed using specific primers for each gene. Normalizer gene: RPL19. The asterisk * above the bars indicates significant differences according to two-way ANOVA (*p* value < 0.05).

Our results indicate that most odorant receptor genes (except for *OR-E*) displayed similar expression levels in parasitoids reared on SA (SA-natal) compared to parasitoids transplanted to SA (originated from AP; Figure 3). In the case of females of *A. ervi* transplanted on AP (originated from SA; Figure 4) compared to females maintained on AP (AP-natal), two ORs showed upregulation (*OR-E* and *OR-J)*, while three ORs were downregulated (*OR-B*, *OR-C*, and *OR-J*). Hence, switching parasitoids from SA to AP had a greater effect on the expression of OR genes, while the expression of OBP genes remained similar in the two conditions (Figure 5).

### 3.4. OBPs and ORs Expression Changes between Field and Caged Parasitoids Reared on Natal and Non-Natal Hosts

We compared gene expression between outbred field (exogamic) and inbred laboratory caged (endogamic) populations of the parasitoid wasp *A. ervi* using the same set of target chemosensation-related genes as above. When gene expression was compared between parasitoids from field and caged populations reared on AP, a slightly but not statistically significant lower expression was observed for OBPs (Figure 5). In the case of ORs, a lower expression was observed for four out of five odorant receptors (for three of them being statistically significant), while only *OR-E* in the inbred population showed a significant higher expression compared to its expression in the field population (Figure 5).

**Figure 6:**
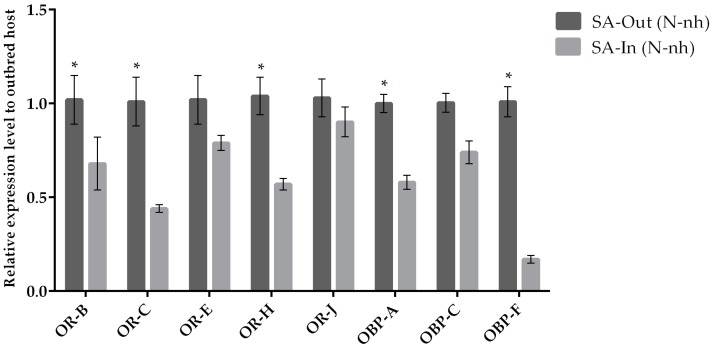
Mean (+/− SE) expression levels of ORs and OBPs from the heads of outbred (SA-exogamic) and inbred (SA-endogamic) *A. ervi* transplanted from the natal host *A. pisum* onto the non-natal host *S. avenae* (SA), measured by RT-qPCR. RT-qPCRs were performed using specific primers for each gene. Normalizer gene: RPL19. The asterisk * above the bars indicates significant differences according to two-way ANOVA (*p* value < 0.05).

Comparisons between outbred (individuals from the field) and inbred (individuals from laboratory cages) parasitoid populations switched to non-natal host SA displayed lower expression levels for both ORs and OBPs (Figure 6).

## 4. Discussion

*A. ervi* has been successfully used for biocontrol of economically relevant aphid species [1], as it has the ability to discriminate between host species (*A. pisum* and *S. avenae*) [30]. In this context, the integration of multiple chemical cues elicits several behaviors that ultimately conduct to the selection and oviposition into a specific aphid host [51,52]. Hence, perception of chemical cues that occur during foraging is crucial for host finding and host recognition, while proteins involved in peripheral olfactory mechanisms (OBPs and ORs) are the first point of neural contact with odorant molecules and chemical cues [47]. Interestingly, it has been proposed that any changes in the expression levels of these families of olfactory genes would have direct effects on downstream odor processing and signal propagation [20,53] and may also play a key role in the case of host fidelity in *A. ervi* [13,30]. Although we know from many examples that insect exposure to novel environments can lead to substantial differences in the transcriptomes of adult individuals [54,55], it is still unclear how much phenotypic plasticity, in terms of expression levels of genes involved in chemical perception mechanisms, is displayed when parasitoids are exploiting different hosts.

In this study, by using RNAseq transcriptomic information, we were able to annotate several chemosensory genes in *A. ervi* and identify genes displaying differential expression levels between parasitoids reared on different hosts. By conducting reciprocal transplant experiments and using quantitative PCR, we found that switching *A. ervi* females to a novel plant–host complex (non-natal host) had significant effects on the transcript abundances of chemosensation-related genes in the offspring of those females, regardless of the natal host (*A. pisum* and *S. avenae*). Surprisingly, we also observed differences in the expression profiles of ORs and OBPs when comparing field and caged parasitoids reared on the same aphid host species. The significantly lower abundance of transcripts measured for these genes might be caused by long-time inbreeding under laboratory conditions (i.e., absence of environmental signals). Thus, it is possible that *A. ervi* inbreeding might disrupt the balance of the highly sensitive and coordinated mechanisms of olfaction, which may explain the loss of host fidelity observed previously [31].

### 4.1. Annotation and in Silico Expression Analysis of Chemosensory Genes in A. ervi

Olfaction and chemosensory perception are key functions for host finding and host recognition. Thus, based on the *A. ervi* transcriptome assembly as a reference [33], we identified a total of 91 unigenes possessing high-sequence identities with chemosensation-related genes, including IRs, ORs, OBPs, CSPs, SNMPs, and Orco. As changes in olfactory sensitivity could be driven by variation in gene expression [28], we also performed in silico gene expression analysis. We found five ORs and three OBPs displaying differential expression levels between *A. ervi* reared on *S. avenae* and reared on *A. pisum* [33]. A possible explanation for this phenomenon is that the expression of these genes is regulated by scent exposure or conditioning, as odorants are handled in a combinatorial fashion [56,57]. Therefore, the exposure to a novel chemical environment during parasitoids’ juvenile development (for example, volatiles from a plant–aphid host complex and host cuticular hydrocarbons) [58] would lead to substantial differences in transcriptional expression levels in adults [54,55] and might be an explanation to the observed changes in gene expression levels [56].

### 4.2. Putative Role of Odorant-Binding Proteins in Parasitoid Wasps

In insects, olfaction is triggered when odorants and other semiochemicals reach the sensillar lymph through pore tubules located in the antenna and bind to OBPs. Then, the odorant–OBP complex is transported through the sensillum lymph to their receptors on olfactory neurons [47]. OBPs are a large family of small, soluble, and highly abundant proteins secreted into the sensory lymph and are thought to provide the first filtering mechanism for semiochemicals, as they are the main proteins involved in the interaction between odorants and membrane-bound ORs [59]. OBPs have been shown to be differentially expressed in subsets of olfactory sensilla in *D. melanogaster* [60], and may contribute to the sensitivity or selectivity of different sensilla types [59]. This has been explained in terms of variable affinities to odorants displayed by OBPs, so that distinct expression patterns for OBP genes suggest odorant selection and triggering of specific olfactory and behavioral responses in insects that impact on host preference [28,41].

The odorant-binding properties of OBPs have been determined for different insect species [41], including the solitary endoparasitoid wasp *Microplitis mediator* [61]. Interestingly, homology searches based on sequences from the endoparasitoid wasp *M. mediator* showed that two of the OBPs analyzed in our study (OBP-A and OBP-F) had high identity values (>40%) with OBP8 and OBP10 from *M. mediator*, while the top blast hit for OBP-C was pheromone-binding protein 1 from *M. mediator*. Functional analysis of OBP8 and OBP10 in *M. mediator* has shown that these genes are expressed mainly in the antennae of adult wasps and can bind a broad range of odorant molecules, including nonane, farnesol, nerolidol, nonanal, β-ionone, acetic ether, and farnesene, with different binding affinities [61]. Additionally, adult parasitoids showed behavioral responses (either attraction or repellence) to these volatiles [61]. The higher expression differences found for OBP-F in parasitoids maintained on *S. avenae* compared to parasitoids maintained on *A. pisum* (regardless of the natal host) may be related to the developmental exposure of *A. ervi* larva to the plant–aphid host complex, as aphid mummies were taken straight from their rearing cages and isolated in Petri dishes. Moreover, exposure to host plant volatiles from infested plants during larval stages of *A. ervi* would induce olfactory responses in the adults [51,62].

### 4.3. Putative Role of Odorant Receptors in Parasitoid Wasps during the Recognition of Their Aphid Hosts

Parasitoid females are attracted to volatiles emitted by aphids and may use them as a host-species recognition mechanism [63]. For instance, E-β-farnesene (EBF) is the alarm pheromone released when aphids are attacked or irritated; EBF is known to attract natural enemies, including the parasitoid wasp *A. ervi* [64]. However, it is unlikely that EBF participates during host acceptance in *A. ervi* due to its lack of specificity, as EBF has been reported in both *S. avenae* and *A. pisum* among other aphid species [65]. Conversely, parasitoids rely on cuticular hydrocarbons (CHCs) present in the aphid exoskeleton. These non-polar lipids serve as species-specific communication cues, among other functions [66], and are composed of a mixture of a few to more than hundreds components of 21–50+ carbon alkanes, alkenes, and branches derivatives [67]. These variations in the CHC profiles appears to be species-specific and a characteristic of an insect species [67,68,69]. Hence, qualitative CHC differences between aphid species would confer parasitoids a barcode to discriminate between hosts at the species level, adjusting their parasitism behavior accordingly (e.g., by triggering specific attack responses) [70,71]. In the case of aphids, *n-*alkanes are the predominant component of CHCs and may include alkenes and their methyl branches derivatives [69,72] but differ among species: in *S. avenae, n-*alkanes range from C_23_ to C_33_, with three predominant compounds being n-Heptacosane (*n*-C_27_, 29%), n-Nonacosane (*n*-C_29_, 27%), and n-Hentriacontane (*n*-C_31_, 10%) [64]. Contrastingly, these three *n-*alkanes are present in different proportions in *A. pisum* (*n*-C_27_, 14%, *n*-C_29_, 48%, *n*-C_31_, 21%) [67].

As host recognition in parasitoid wasps is achieved after antennal contact with kairomones and chemical cues located on the cuticle of insects [73], it is expected that ORs contribute to the detection and discrimination of different CHCs. This is the case for the Indian jumping ant *Harpegnathos saltator* (Hymenoptera: Formicidae), where several ORs are narrowly tuned to specific CHCs [74]. As current evidence suggests that OR expression is amenable to modulation by scent conditioning [56], *A. ervi* parasitoids reared on their natal host are not expected to respond to volatiles which they have not been previously exposed to or experienced (i.e., volatiles derived from the non-natal plant–host complex). This is because OR-coding genes might change their regulation and expression as a response to long exposures to specific environments, for example, during the developmental time into the body of the aphid host, which should modify the offspring oviposition behavior [75].

In our study, a significant downregulation was observed for four out of five odorant receptors (*OR-B, OR-E, OR-H*, and *OR-J*) in parasitoids transplanted onto *S. avenae* compared with parasitoids reared on the natal host *A. pisum*. Furthermore, homology searches using these four ORs as queries in BLASTx alignments against *Drosophila* spp. found their corresponding homolog sequences (*OR9a*, *OR43a*, *OR13a*, and *OR85d*, respectively; Table 3) and their odorant-response profiles [45] (Table 3). These odorant receptors show high responsivity to several volatiles emitted from the *A. pisum*–*Vicia faba* host plant complex [71,73,76], which are known to attract aphid natural enemies, including the parasitoid wasp *A. ervi* [77,78]. These volatiles include 3-hydroxy-2-butanone, a volatile present in the excreted honeydew of *A. pisum* when feeding on *Vicia faba*, (*Z*)-3-hexen-1-ol, which is one of the most abundant compounds found in the volatile blends emitted by *V. faba* plants when infested with *A. pisum,* and 1-Octen-3-ol, a volatile emitted by *V. faba* plants in response to herbivore walking activity [71,79]. Interestingly, *OR85d* shows response to 6-methyl-5-hepten-2-one [45], which is one of the most attractive volatiles for *A. ervi* females and is found in the headspace of *V. faba* plants infested with *A. pisum*. However, the release of this compound is not induced by other aphids like the black bean aphid *Aphis fabae* (Hemiptera: Aphididae), a non-suitable aphid host for *A. ervi* [77]. Hence, our results suggest that *A. ervi* reared on *A. pisum* are able to display plasticity for the expression of ORs when transplanted to a new aphid–plant complex (e.g., *S. avenae*–barley). This downregulation observed in *A. ervi* transplanted from *A. pisum* to *S. avenae* could be a consequence of a reduced exposure to volatiles from the interaction between *A. pisum* and broad bean and suggests that the expression of chemosensory genes is indeed affected by the exposure to plant volatiles, as reported for other insect species [80]. In the case of *A. ervi* transplanted from *S. avenae* onto *A. pisum*, only *OR-H* showed a significant upregulation, while the other ORs showed no variation. This imply that *OR-H* display changes in gene expression in response to new chemical cues, thus suggesting that *A. ervi* originated from a cereal aphid display a narrow plasticity in terms of ORs gene expression compared to parasitoids originated from a legume aphid.

### 4.4. Expression Changes in OBPs and ORs between Field and Caged A. ervi Populations: Implications for Loss of Host Fidelity

Chemosensory mechanisms play a key role in insect host location and host discrimination [18]. However, under laboratory rearing conditions, a loss of sensitivity and reduced variation in olfactory responses toward host volatiles may be observed [81]. This may have a significant impact on host fidelity, as a poor discrimination of specific cues from a blend of volatiles can restrict or even impair olfaction in insects [82]. Previous reports indicate that inbred *A. ervi* parasitoids rapidly accept aphids with no true selection of hosts, regardless of the natal host from which the parasitoids originated [31]. Hence, it is likely that the inbreeding caused by several generations of confined rearing conditions negatively impact on the regulation of chemosensory genes, with respect to the parasitoids’ “wild” counterparts sampled from the field, even if the parasitoids are kept on the same natal aphid–plant host complex (*A. pisum*) or are transplanted onto a novel aphid–plant host complex (*S. avenae*).

While field populations of *A. ervi* were collected from alfalfa crops and maintained in an *A. pisum*–*Vicia faba* system for two generations, caged populations also sampled from alfalfa were maintained for over 2 years (>75 generations) in that same aphid–plant system [31]. As the highly inbred laboratory populations had not been exposed to the variety of volatiles emitted from other plants and animals in nature, this may have led to an inaccurate sensory processing due to the lack of modulation by scent conditioning [56]. Furthermore, exposure to *V. faba* for several generations might explain the higher *OR-E* expression and the reduced expression of other chemosensory genes observed when field and caged populations were compared.

The reduced expression of ORs and OBPs observed in inbred *A. ervi* populations would explain their changes in host preference behavior [31], where a rapid host aphid acceptance and poor discrimination (loss of host fidelity) were observed, compared to their wild counterparts [30,31,83]. Interestingly, behavioral changes (e.g., weaker attraction to host plant volatiles) have also been reported for other predatory insects reared under confined laboratory conditions [84]. It is also noteworthy that the synthesis of extremely high quantities of OBPs and ORs requires large amounts of energy, which cannot be obtained without a fitness cost. This is particularly true in insects, which often have critical energy budgets [85,86]. However, rearing under highly homogenous and stable laboratory conditions for several generations with plentiful resources (food and hosts readily available) may relax the mechanisms in charge of keeping an optimal, “ready-to-use” olfaction system. Hence, under these homogenous conditions, parasitoids may shut down the expression of certain OBPs and ORs, losing the fine-tuning ability to discriminate among potential aphid hosts.

### 4.5. Implications for Biological Control and Final Remarks

The loss of sensitivity and discrimination ability of the olfaction system in a laboratory-reared parasitoid wasp can have undesirable effects on the efficiency of biological control, preventing the identification of a specific target pest in agroecosystems [87]. Hence, the downregulation of ORs and OBPs observed in inbred populations may explain their changes in host preference behavior related to a more rapid aphid acceptance and poor host discrimination compared to wasps from the field [30,31,83]. Moreover, the efficiency of biological control can also be threatened by a loss of fitness related to a biased production of males in the offspring of inbred parasitoids [31]. Therefore, the mass-reared production of parasitoid wasps for biological control programs should be carefully managed before their release at the farm scale, as the founder effect, genetic drift, and inbreeding depression can provoke profound and unpredictable changes in behavioral, physiological, and olfactory traits of relatively small caged populations reared on the same aphid–plant complex. Further studies on the molecular basis of host fidelity can shed light on how changes in olfaction sensitivity underpin changes in host preference and host fidelity. These may involve the study of the electrophysiological responses to specific volatiles when parasitoid wasps face natal and non-natal hosts and the study of the behavioral responses when specific *OR* genes are silenced, in order to verify whether host selection in *A. ervi* is actually based on changes in gene expression rather than on genetic differences.

## Figures and Tables

**Table 1:** Chemosensory genes annotated from the *Aphidius ervi* reference transcriptome assembly. Transcripts displaying significant differential expression patterns between *A. ervi–Acyrthosiphon pisum* (AP) and *A. ervi–Sitobion avenae* (SA) are marked in bold characters and with *. FDR: false discovery rate.

Transcript ID	Sequence Description	Higher in	Log2-Fold Change	FDR-Adjusted *p* Value
TR35948|c0_g1_i1	general odorant-binding protein 56a-like	Ae-AP	1.58	0.17
TR39104|c3_g3_i1	general odorant-binding protein 69a	Ae-AP *	3.36	0.00023
TR42476|c0_g1_i1	general odorant-binding protein 69a-like	Ae-AP	1.91	0.07
TR35957|c0_g1_i3	general odorant-binding protein 71 isoform X1	Ae-AP	2.27	0.07
TR10701|c0_g1_i1	general odorant-binding protein 83a-like	Ae-AP *	4.40	0.00038
TR12460|c0_g3_i1	odorant receptor 10a-like	Ae-AP	3.30	0.03
TR20850|c0_g1_i1	odorant receptor 13a-like	Ae-AP	1.64	0.22
TR22319|c1_g1_i1	odorant receptor 13a-like	Ae-AP	6.55	0.14
TR2742|c0_g1_i2	odorant receptor 13a-like	Ae-AP *	4.13	0.0023
TR29575|c0_g1_i1	odorant receptor 13a-like	Ae-AP	0.97	0.67
TR30006|c0_g1_i1	odorant receptor 13a-like	Ae-AP	2.15	0.29
TR30197|c0_g2_i1	odorant receptor 13a-like	Ae-AP	3.18	0.04
TR39962|c0_g1_i1	odorant receptor 13a-like	Ae-AP	3.29	0.08
TR41237|c0_g2_i1	odorant receptor 13a-like	Ae-AP	1.66	0.27
TR48968|c0_g1_i2	odorant receptor 13a-like	Ae-AP *	3.24	0.0034
TR52641|c0_g1_i2	odorant receptor 13a-like	Ae-AP	1.19	0.43
TR9036|c4_g1_i7	odorant receptor 13a-like	Ae-AP	1.61	0.50
TR7457|c0_g1_i1	odorant receptor 13a-like isoform X1	Ae-AP *	8.37	0.00032
TR41237|c0_g1_i1	odorant receptor 13a-like isoform X2	Ae-AP	0.17	0.92
TR52641|c0_g1_i3	odorant receptor 13a-like isoform X2	Ae-AP *	3.58	0.0037
TR19916|c0_g1_i3	odorant receptor 13a-like, partial	Ae-AP	0.30	0.72
TR54924|c0_g1_i1	odorant receptor 22c-like	Ae-AP	1.30	0.50
TR41029|c0_g1_i1	odorant receptor 24a-like	Ae-AP	1.03	0.57
TR52071|c2_g1_i1	odorant receptor 2a-like	Ae-AP	0.83	0.67
TR8156|c0_g1_i2	odorant receptor 2a-like	Ae-AP	2.06	0.15
TR52486|c1_g1_i1	odorant receptor 30a-like	Ae-AP	1.56	0.48
TR36608|c0_g2_i2	odorant receptor 46a, isoform A-like isoform X2	Ae-AP	1.37	0.58
TR1120|c1_g1_i1	odorant receptor 4-like	Ae-AP	0.96	0.60
TR46910|c0_g1_i1	odorant receptor 4-like	Ae-AP	7.05	0.06
TR42319|c6_g4_i1	odorant receptor 67a-like	Ae-AP	1.06	0.48
TR1484|c4_g2_i5	odorant receptor 67c-like	Ae-AP	0.56	0.67
TR20646|c1_g1_i1	odorant receptor 67c-like	Ae-AP	3.63	0.03
TR36143|c1_g1_i1	odorant receptor 71a	Ae-AP	0.11	1.00
TR42698|c0_g1_i1	odorant receptor 85c-like isoform X1	Ae-AP	1.65	0.53
TR9036|c4_g1_i6	odorant receptor 85d	Ae-AP	2.86	0.08
TR19916|c0_g1_i5	odorant receptor 98b	Ae-AP	4.49	0.04
TR54734|c0_g1_i1	odorant receptor 9a-like isoform X1	Ae-AP	0.37	0.83
TR8264|c23_g1_i1	odorant receptor coreceptor	Ae-AP	0.87	0.54
TR3968|c1_g1_i1	odorant receptor Or1-like	Ae-AP	0.18	0.89
TR13645|c0_g1_i2	odorant receptor Or1-like isoform X2	Ae-AP	1.00	0.52
TR55175|c0_g1_i1	chemosensory protein 3	Ae-AP	1.43	0.40
TR53809|c1_g1_i1	ionotropic receptor 25a.1	Ae-AP	0.22	0.95
TR28446|c0_g1_i1	ionotropic receptor 76b	Ae-AP	0.61	0.81
TR15279|c0_g1_i1	odorant receptor 13a-like	Ae-AP	1.19	0.70
TR7457|c1_g1_i1	odorant receptor 13a-like	Ae-AP	1.68	0.19
TR9036|c4_g1_i10	odorant receptor 13a-like	Ae-AP	0.39	0.77
TR22647|c0_g1_i1	odorant receptor 23	Ae-AP	5.90	0.21
TR52071|c0_g2_i1	odorant receptor 28	Ae-AP	2.46	0.19
TR656|c0_g1_i1	odorant receptor 2a isoform X1	Ae-AP	2.59	0.15
TR1484|c4_g6_i2	odorant receptor 33a-like isoform x2	Ae-AP	0.97	0.73
TR3968|c0_g1_i4	odorant receptor 35	Ae-AP	0.63	0.66
TR44731|c1_g1_i1	odorant receptor 39	Ae-AP	1.20	0.38
TR44731|c1_g1_i2	odorant receptor 39	Ae-AP	2.07	0.08
TR44731|c1_g1_i4	odorant receptor 39	Ae-AP	0.60	0.49
TR52645|c3_g1_i9	odorant receptor 4-like	Ae-AP	0.60	0.51
TR42319|c6_g3_i1	odorant receptor 67a-like	Ae-AP	2.50	0.32
TR48050|c0_g2_i1	odorant receptor 85e	Ae-AP	2.45	0.03
TR48683|c0_g1_i1	odorant receptor or1-like	Ae-AP *	9.01	0.00031
TR4899|c1_g1_i1	odorant-binding protein 1	Ae-AP	1.31	0.26
TR9442|c0_g1_i1	odorant-binding protein 10	Ae-AP	1.14	0.36
TR29385|c0_g4_i8	olfactory receptor 11	Ae-AP	0.17	0.96
TR48827|c0_g1_i1	sensory neuron membrane protein 1	Ae-AP	1.34	0.24
TR33912|c0_g1_i1	chemosensory protein 5	Ae-SA	1.24	0.37
TR20258|c0_g1_i1	general odorant-binding protein 83a-like	Ae-SA	0.00	1.00
TR46958|c0_g1_i1	general odorant-binding protein 83a-like	Ae-SA *	7.62	0.0021
TR14273|c0_g1_i1	general odorant-binding protein 72-like	Ae-SA	2.99	0.30
TR1120|c0_g1_i1	odorant receptor 13a-like	Ae-SA	0.03	1.00
TR1120|c2_g1_i1	odorant receptor 13a-like	Ae-SA	6.24	0.12
TR24336|c0_g1_i1	odorant receptor 13a-like	Ae-SA	0.46	0.81
TR53167|c0_g1_i1	odorant receptor 13a-like	Ae-SA	0.58	0.66
TR5540|c0_g2_i1	odorant receptor 13a-like	Ae-SA	3.21	0.53
TR7457|c0_g1_i2	odorant receptor 13a-like	Ae-SA	4.40	0.50
TR30703|c0_g1_i1	odorant receptor 2a-like	Ae-SA	3.12	0.60
TR53618|c1_g1_i1	odorant receptor 33a-like isoform X2	Ae-SA	0.16	0.80
TR5622|c11_g2_i2	odorant receptor 46a, isoform A-like	Ae-SA	0.85	0.58
TR36608|c0_g1_i1	odorant receptor 46a, isoform A-like isoform X1	Ae-SA	0.74	0.83
TR36608|c0_g2_i3	odorant receptor 46a, isoform A-like isoform X1	Ae-SA	0.87	0.67
TR24403|c0_g1_i1	odorant receptor 49b-like	Ae-SA	0.59	0.90
TR39895|c0_g1_i1	odorant receptor 67c-like	Ae-SA	0.66	0.94
TR15590|c0_g1_i1	odorant receptor 9a-like	Ae-SA	3.41	0.62
TR35582|c0_g1_i1	odorant receptor Or1-like	Ae-SA	0.71	0.79
TR3933|c3_g2_i1	odorant receptor 33a-like isoform X2	Ae-SA	0.17	0.49
TR13230|c0_g1_i1	odorant receptor 38	Ae-SA	0.11	1.00
TR19916|c0_g1_i2	odorant receptor 43	Ae-SA	1.29	0.73
TR3933|c4_g2_i5	odorant receptor 67c-like	Ae-SA	0.82	0.60
TR9036|c4_g1_i5	odorant receptor 85d	Ae-SA	3.83	0.41
TR22375|c0_g1_i1	odorant receptor isoform a-like	Ae-SA	1.47	0.64
TR26430|c0_g1_i1	odorant receptor isoform a-like isoform X1	Ae-SA	0.33	0.86
TR33617|c0_g1_i1	odorant receptor or2-like isoform X1	Ae-SA	0.35	0.79
TR46436|c0_g1_i1	odorant receptor Or3h, partial	Ae-SA	1.69	0.87

**Table 2:** Nucleotide sequences of the primers employed for qPCR in this study. The listed primers for RPL19, used as a normalizer gene for qPCR analysis in *A. ervi*, are the same used by Colinet et al. 2014. OBP: odorant-binding protein, OR: odorant receptor.

Transcript ID	Amplicon ID	Forward Primer (5′-3′)	Reverse Primer (5′-3′)	TM (°C)
TR10701|c0_g1_i1	OBP-A	AGCAGTTCAATCAATTCAAG	TTCAAGTAGTCATATAGTTGGT	58.3
TR39104|c3_g3_i1	OBP-C	TTGAAGTTGAAATGTTGGTT	CACATATCAGGTCTTGTTTG	58.0
TR46958|c0_g1_i1	OBP-F	TACGATATTTACCATACAGCAT	TAGTGGAACAATTTGAAGAAC	58.7
TR2742|c0_g1_i2	OR-B	ACAACAGACAATGTGTATTC	AGTATAAATGGTCCTGCTAAT	57.8
TR48683|c0_g1_i1	OR-C	GCAATTTGTTACGGACTATT	GTTGTTTACTGTCACACATT	58.1
TR48968|c0_g1_i2	OR-E	TCAACAAATTCCTCCTTACA	ATACAATATGGTGGCGATAA	58.1
TR7457|c0_g1_i1	OR-H	GTCATTATTCACAGTTGGATT	GTATCAAGAGCAACAACAATA	58.0
TR52641|c0_g1_i3	OR-J	TTGATGGTGATAATGGTAAGA	CACTTGACGATATAATGACAA	57.8
JAC59129.1 †	RPL19	ATCAAGCTGAAGCTCGTCGT	TGCAGCTGCTTCATCTTCAC	56.6

† indicates the coding sequence of the ribosomal protein L19 from *A. ervi* available at NCBI GenBank.

**Table 3:** Odorant receptor and odorant-binding protein homologs from *Drosophila spp.* found in *A. ervi* using BLAST. The odorants eliciting the responses are shown.

Transcript ID	Amplicon ID	Best *Drosophila* Hit	Response/Tuning To	Reference
TR10701|c0_g1_i1	OBP-A	Odorant-binding protein *Lush*	ο (*Z*)-11-octadecenyl acetate ο 11-cis vaccenyl acetate	Fan et al. 2011 [41]
TR39104|c3_g3_i1	OBP-C	Odorant-binding protein 83a	ο l-carvone ο Citral	Swarup et al. 2011 [42]
TR46958|c0_g1_i1	OBP-F †	Odorant-binding protein 56e	ο Octanoic acid ο Hexanoic acid	Dworkin & Jones 2009 [43]
TR2742|c0_g1_i2	OR-B	Odorant receptor 9a	ο 3-hydroxy-2-butanone ο 2,3-butadeniol ο 2-pentanol	Saberi & Seyed-Allaei 2016 [44]
TR48683|c0_g1_i1	OR-C	Odorant receptor 82a	ο Geranyl acetate ο (2R)-hexan-2-ol ο Citral	Münch & Galizia 2016 [45]
TR48968|c0_g1_i2	OR-E	Odorant receptor 43a	ο Z3-hexenol ο 1-hexanol ο Cyclohexanol ο 1-octen-3-ol ο 2-pentanol	Münch & Galizia 2016 [45]
TR7457|c0_g1_i1	OR-H	Odorant receptor 13a	ο 1-octen-3-ol ο 2-heptanol ο 2-exanol ο 3-octanol	Münch & Galizia 2016 [45]
TR52641|c0_g1_i3	OR-J	Odorant receptor 85d	ο Ethyl pentanoate ο 2-heptanone-6-methyl-5-hepten-2-none	Münch & Galizia 2016 [45]

† indicates transcripts with higher expression levels in *A. ervi*–SA as indicated by previous transcriptomic analysis.

## Supplementary Materials

The following are available online at https://www.mdpi.com/2075-4450/10/11/397/s1, Figure S1: Reciprocal transplant experiment of exogamic populations of *Aphidius ervi* (panels a, b, c, d) and comparison with endogamic populations on the same hosts (Sepúlveda et al. 2017a; panels e, f).

## References

[1] Godfray H.C.J. (1994). Parasitoids: Behavioral and Evolutionary Ecology.

[2] Le Ralec A., Anselme C., Outreman Y., Poirié M., Van Baaren J., Le Lann C.C., Van Alphen J.J.M. (2010). Evolutionary ecology of the interactions between aphids and their parasitoids. C. R. Biol..

[3] Simpson M., Gurr G.M., Simmons A.T., Wratten S.D., James D.G., Leeson G., Nicol H.I., Orre-Gordon G.U.S. (2011). Attract and reward: Combining chemical ecology and habitat manipulation to enhance biological control in field crops. J. Appl. Ecol..

[4] Starý P. (1993). The fate of released parasitoids (Hymenoptera: Braconidae, Aphidiinae) for biological control of aphids in Chile. Bull. Entomol. Res..

[5] Henry L.M., May N., Acheampong S., Gillespie D.R., Roitberg B.D. (2010). Host-adapted parasitoids in biological control: Does source matter?. Ecol. Appl..

[6] Stilmant D., Bellinghen C., Hance T., Boivin G. (2008). Host specialization in habitat specialists and generalists. Oecologia.

[7] Daza-Bustamante P., Fuentes-Contreras E., Niemeyer H.M. (2003). Acceptance and suitability of *Acyrthosiphon pisum* and *Sitobion avenae* as hosts of the aphid parasitoid *Aphidius ervi* (Hymenoptera: Braconidae). Eur. J. Entomol..

[8] Gerding M., Figueroa A. (1989). Progeny reduction of *Sitobion avenae* Fab (Homoptera: Aphididae) by *Aphidius ervi* (Hymenoptera: Aphidiidae). Agric. Técnica.

[9] Pan M.Z., Liu T.X. (2014). Suitability of three aphid species for *Aphidius gifuensis* (Hymenoptera: Braconidae): Parasitoid performance varies with hosts of origin. Biol. Control.

[10] Nouhuys S., Via S. (1999). Natural selection and genetic differentiation of behaviour between parasitoids from wild and cultivated habitats. Heredity.

[11] Jones T.S., Bilton A.R., Mak L., Sait S.M. (2015). Host switching in a generalist parasitoid: Contrasting transient and transgenerational costs associated with novel and original host species. Ecol. Evol..

[12] Jones T.S., Godfray H.C.J., van Veen F.J.F. (2009). Resource Competition and Shared Natural Enemies in Experimental Insect Communities. Oecologia.

[13] Henry L.M., Roitberg B.D., Gillespie D.R. (2008). Host-range evolution in *Aphidius* parasitoids: Fidelity, virulence and fitness trade-offs on an ancestral host. Evolution.

[14] Spitzer B.W. (2004). Maternal Effects in the Soft Scale Insect *Saisetia coffeae* (Hemiptera: Coccidae). Evolution.

[15] Hoedjes K.M., Kruidhof H.M., Huigens M.E., Dicke M., Vet L.E.M., Smid H.M. (2011). Natural variation in learning rate and memory dynamics in parasitoid wasps: Opportunities for converging ecology and neuroscience. Proc. Biol. Sci..

[16] Davis J.M., Stamps J.A. (2004). The effect of natal experience on habitat preferences. Trends Ecol. Evol..

[17] Gols R., Veenemans C., Potting R.P.J., Smid H.M., Dicke M., Harvey J. a., Bukovinszky T. (2012). Variation in the specificity of plant volatiles and their use by a specialist and a generalist parasitoid. Anim. Behav..

[18] Suh E., Bohbot J.D., Zwiebel L.J. (2014). Peripheral olfactory signaling in insects. Curr. Opin. Insect Sci..

[19] Hilker M., McNeil J. (2008). Chemical and behavioral ecology in insect parasitoids: How to behave optimally in a complex odorous environment. Behavioral Ecology of Insect Parasitoids.

[20] Claudianos C., Lim J., Young M., Yan S., Cristino A.S., Newcomb R.D., Gunasekaran N., Reinhard J. (2014). Odor memories regulate olfactory receptor expression in the sensory periphery. Eur. J. Neurosci..

[21] Linz J., Baschwitz A., Strutz A., Dweck H.K.M., Sachse S., Hansson B.S., Stensmyr M.C. (2013). Host plant-driven sensory specialization in *Drosophila erecta*. Proc. R. Soc. B Biol. Sci..

[22] Rehman A., Powell W. (2010). Host selection behaviour of aphid parasitoids (Aphidiidae: Hymenoptera). J. Plant Breed. Crop Sci..

[23] Wajnberg E., Colazza S. (2013). Chemical Ecology of Insect Parasitoids.

[24] Wang Q., Gu H., Dorn S. (2003). Selection on olfactory response to semiochemicals from a plant-host complex in a parasitic wasp. Heredity.

[25] Gadenne C., Barrozo R.B., Anton S. (2016). Plasticity in insect olfaction: To smell or not to smell?. Annu. Rev. Entomol..

[26] Arya G.H., Magwire M.M., Huang W., Serrano-Negron Y.L., Mackay T.F.C., Anholt R.R.H. (2015). The genetic basis for variation in olfactory behavior in *Drosophila melanogaster*. Chem. Senses.

[27] Zhou S., Stone E.A., Mackay T.F.C., Anholt R.R.H. (2009). Plasticity of the chemoreceptor repertoire in *Drosophila melanogaster*. PLoS Genet..

[28] Glaser N., Gallot A., Legeai F., Harry M., Kaiser L., Le Ru B., Calatayud P.A., Jacquin-Joly E. (2015). Differential expression of the chemosensory transcriptome in two populations of the stemborer *Sesamia nonagrioides*. Insect Biochem. Mol. Biol..

[29] Smith G., Fang Y., Liu X., Kenny J., Cossins A.R., de Oliveira C.C., Etges W.J., Ritchie M.G. (2013). Transcriptome-wide expression variation associated with environmental plasticity and mating success in cactophilic *Drosophila mojavensis*. Evolution.

[30] Zepeda-Paulo F.A., Ortiz-Martínez S.A., Figueroa C.C., Lavandero B. (2013). Adaptive evolution of a generalist parasitoid: Implications for the effectiveness of biological control agents. Evol. Appl..

[31] Sepúlveda D.A., Zepeda-Paulo F., Ramírez C.C., Lavandero B., Figueroa C.C. (2017). Loss of host fidelity in highly inbred populations of the parasitoid wasp *Aphidius ervi* (Hymenoptera: Braconidae). J. Pest Sci..

[32] Starý P. (1995). The Aphidiidae of Chile (Hymenoptera, Ichneumonoidea, Aphidiidae). Dtsch. Entomol. Z..

[33] Ballesteros G.I., Gadau J., Legeai F., Gonzalez-Gonzalez A., Lavandero B., Simon J.-C., Figueroa C.C. (2017). Expression differences in *Aphidius ervi* (Hymenoptera: Braconidae) females reared on different aphid host species. PeerJ.

[34] Dennis A.B., Patel V., Oliver K.M., Vorburger C. (2017). Parasitoid gene expression changes after adaptation to symbiont-protected hosts. Evolution.

[35] Sepúlveda D.A., Zepeda-Paulo F., Ramírez C.C., Lavandero B., Figueroa C.C. (2017). Diversity, frequency, and geographic distribution of facultative bacterial endosymbionts in introduced aphid pests. Insect Sci..

[36] Oliver K.M., Martinez A.J. (2014). How resident microbes modulate ecologically-important traits of insects. Curr. Opin. Insect Sci..

[37] Vorburger C. (2014). The evolutionary ecology of symbiont-conferred resistance to parasitoids in aphids. Insect Sci..

[38] Langmead B., Trapnell C., Pop M., Salzberg S.L. (2009). Ultrafast and memory-efficient alignment of short DNA sequences to the human genome. Genome Biol..

[39] Li B., Dewey C.N. (2011). RSEM: Accurate transcript quantification from RNA-Seq data with or without a reference genome. BMC Bioinform..

[40] Benjamini Y., Hochberg Y. (1995). Controlling the False Discovery Rate: A Practical and Powerful Approach to Multiple Testing. J. R. Stat. Soc. Ser. B.

[41] Fan J., Francis F., Liu Y., Chen J.L., Cheng D.F. (2011). An overview of odorant-binding protein functions in insect peripheral olfactory reception. Genet. Mol. Res..

[42] Swarup S., Williams T.I., Anholt R.R.H. (2011). Functional dissection of Odorant binding protein genes in *Drosophila melanogaster*. Genes Brain Behav..

[43] Dworkin I., Jones C.D. (2009). Genetic Changes Accompanying the Evolution of Host Specialization in *Drosophila sechellia*. Genetics.

[44] Saberi M., Seyed-allaei H. (2016). Odorant receptors of *Drosophila* are sensitive to the molecular volume of odorants. Sci. Rep..

[45] Münch D., Galizia C.G. (2016). DoOR 2.0—Comprehensive Mapping of *Drosophila melanogaster* Odorant Responses. Sci. Rep..

[46] Jin S., Zhou X., Gu F., Zhong G., Yi X. (2017). Olfactory Plasticity: Variation in the Expression of Chemosensory Receptors in *Bactrocera dorsalis* in Different Physiological States. Front. Physiol..

[47] Leal W.S. (2013). Odorant Reception in Insects: Roles of Receptors, Binding Proteins, and Degrading Enzymes. Annu. Rev. Entomol..

[48] Wang R., Li F., Zhang W., Zhang X., Qu C., Tetreau G., Sun L., Luo C., Zhou J. (2017). Identification and expression profile analysis of odorant binding protein and chemosensory protein genes in *Bemisia tabaci* MED by head transcriptome. PLoS ONE.

[49] Colinet D., Anselme C., Deleury E., Mancini D., Poulain J., Azéma-Dossat C., Belghazi M., Tares S., Pennacchio F., Poirié M. (2014). Identification of the main venom protein components of *Aphidius ervi*, a parasitoid wasp of the aphid model *Acyrthosiphon pisum*. BMC Genom..

[50] Livak K.J., Schmittgen T.D. (2001). Analysis of Relative Gene Expression Data Using Real-Time Quantitative PCR and the 2−ΔΔCT Method. Methods.

[51] Takemoto H., Kainoh Y., Takabayashi J. (2011). Learning of plant volatiles by aphid parasitoids: Timing to learn. J. Plant Interact..

[52] Powell W., Wright A.F. (1988). The abilities of the aphid parasitoids *Aphidius ervi* Haliday and *A. rhopalosiphi* De Stefani Perez (Hymenoptera: Braconidae) to transfer between different known host species and the implications for the use of alternative hosts in pest control strategies. Bull. Entomol. Res..

[53] Kopp A., Barmina O., Hamilton A.M., Higgins L., McIntyre L.M., Jones C.D. (2008). Evolution of Gene Expression in the *Drosophila* Olfactory System. Mol. Biol. Evol..

[54] Berens A.J., Hunt J.H., Toth A.L. (2015). Nourishment level affects caste-related gene expression in *Polistes* wasps. BMC Genom..

[55] Schrader L., Simola D.F., Heinze J., Oettler J. (2015). Sphingolipids, transcription factors, and conserved toolkit genes: Developmental plasticity in the ant cardiocondyla obscurior. Mol. Biol. Evol..

[56] Kang Z.-W., Tian H.-G., Liu F.-H., Liu X., Jing X.-F., Liu T.-X. (2017). Identification and expression analysis of chemosensory receptor genes in an aphid endoparasitoid *Aphidius gifuensis*. Sci. Rep..

[57] Pareja M., Mohib A., Birkett M.A., Dufour S., Glinwood R.T. (2009). Multivariate statistics coupled to generalized linear models reveal complex use of chemical cues by a parasitoid. Anim. Behav..

[58] Villagra C.A., Pennacchio F., Niemeyer H.M. (2007). The effect of larval and early adult experience on behavioural plasticity of the aphid parasitoid *Aphidius ervi* (Hymenoptera, Braconidae, Aphidiinae). Naturwissenschaften.

[59] Tunstall N.E., Warr C.G. (2012). Chemical communication in insects: The peripheral odour coding system of drosophila melanogaster. Adv. Exp. Med. Biol..

[60] Larter N.K., Sun J.S., Carlson J.R. (2016). Organization and function of *Drosophila* odorant binding proteins. Elife.

[61] Li K., Wang S., Zhang K., Ren L., Ali A., Zhang Y., Zhou J., Guo Y. (2014). Odorant Binding Characteristics of Three Recombinant Odorant Binding Proteins in *Microplitis mediator* (Hymenoptera: Braconidae). J. Chem. Ecol..

[62] Gutiérrez-Ibáñez C., Villagra C.A., Niemeyer H.M. (2007). Pre-pupation behaviour of the aphid parasitoid *Aphidius ervi* (Haliday) and its consequences for pre-imaginal learning. Naturwissenschaften.

[63] Poppy G.M., Powell W., Pennacchio F. (1997). Aphid parasitoid responses to semiochemicals—Genetic, conditioned or learnt?. Entomophaga.

[64] Cui L.-L., Francis F., Heuskin S., Lognay G., Liu Y.-J., Dong J., Chen J.-L., Song X.-M., Liu Y. (2012). The functional significance of E-β-Farnesene: Does it influence the populations of aphid natural enemies in the fields?. Biol. Control.

[65] Francis F., Vandermoten S., Verheggen F., Lognay G., Haubruge E. (2005). Is the E-b-farnesene the only volatile terpenoid in aphids?. Jen.

[66] Howard R.W., Blomquist G.J. (2004). Ecological, behavioral and biochemical aspects of insect hydrocarbons. Annu. Rev. Entomol..

[67] Chen N., Fan Y., Li X., Liu T. Dynamic cuticular hydrocarbon profiles of the pea aphid, *Acyrthosiphon pisum*. Proceedings of the 5th International Symposium on Insect Physiology, Biochemistry and Molecular Biology.

[68] Lockey K.H. (1988). Lipids of the insect cuticle: Origin, composition and function. Comp. Biochem. Physiol. Part B Biochem..

[69] Muratori F., Hance T., Lognay G.C. (2008). Chemical characterization of cuticular extracts of *Sitobion avenae* (Hemiptera: Aphididae). Ann. Entomol. Soc. Am..

[70] Hatano E., Kunert G., Michaud J.P., Weisser W.W. (2008). Chemical cues mediating aphid location by natural enemies. Eur. J. Entomol..

[71] Muratori F., Le Ralec A., Lognay G., Hance T. (2006). Epicuticular factors involved in host recognition for the aphid parasitoid *Aphidius rhopalosiphi*. J. Chem. Ecol..

[72] Brey P.T., Ohayon H., Lesourd M., Castex H., Roucache J., Latge J.P. (1985). Ultrastructure and chemical composition of the outer layers of the cuticle of the pea aphid *Acyrthosiphon pisum* (Harris). Comp. Biochem. Physiol. Part A Physiol..

[73] Le Ralec A., Curty C., Wajnberg É. (2005). Inter-specific variation in the reactive distance of different aphid-parasitoid associations: Analysis from automatic tracking of the walking path. Appl. Entomol. Zool..

[74] Pask G.M., Slone J.D., Millar J.G., Das P., Moreira J.A., Zhou X., Bello J., Berger S.L., Bonasio R., Desplan C. (2017). Specialized odorant receptors in social insects that detect cuticular hydrocarbon cues and candidate pheromones. Nat. Commun..

[75] Wang Y., Chen Q., Guo J., Li J., Wang J., Wen M., Zhao H., Ren B. (2017). Molecular basis of peripheral olfactory sensing during oviposition in the behavior of the parasitic wasp *Anastatus japonicus*. Insect Biochem. Mol. Biol..

[76] Leroy P.D., Sabri A., Heuskin S., Thonart P., Lognay G., Verheggen F.J., Francis F., Brostaux Y., Felton G.W., Haubruge E. (2011). Microorganisms from aphid honeydew attract and enhance the efficacy of natural enemies. Nat. Commun..

[77] Du Y.J., Poppy G.M., Powell W., Pickett J.A., Wadhams L.J., Woodcock C.M. (1998). Identification of semiochemicals released during aphid feeding that attract parasitoid *Aphidius ervi*. J. Chem. Ecol..

[78] Sasso R., Iodice L., Woodcock C.M., Pickett J.A., Guerrieri E. (2009). Electrophysiological and behavioural responses of *Aphidius ervi* (Hymenoptera: Braconidae) to tomato plant volatiles. Chemoecology.

[79] Frati F., Cusumano A., Conti E., Colazza S., Peri E., Guarino S., Martorana L., Romani R., Salerno G. (2017). Foraging behaviour of an egg parasitoid exploiting plant volatiles induced by pentatomids: The role of adaxial and abaxial leaf surfaces. PeerJ.

[80] Wan X., Qian K., Du Y. (2015). Synthetic pheromones and plant volatiles alter the expression of chemosensory genes in *Spodoptera exigua*. Sci. Rep..

[81] Nielsen M.C., Worner S.P., Rostás M., Chapman R.B., Butler R.C., de Kogel W.J., Teulon D.A.J. (2015). Olfactory responses of western flower thrips (*Frankliniella occidentalis*) populations to a non-pheromone lure. Entomol. Exp. Appl..

[82] Webster B., Qvarfordt E., Olsson U., Glinwood R. (2013). Different roles for innate and learnt behavioral responses to odors in insect host location. Behav. Ecol..

[83] Daza-Bustamante P., Fuentes-Contreras E., Rodriguez L.C., Figueroa C.C., Niemeyer H.M. (2002). Behavioural differences between *Aphidius ervi* populations from two tritrophic systems are due to phenotypic plasticity. Entomol. Exp. Appl..

[84] Dicke M., Schütte C., Dijkman H. (2000). Change in behavioral response to herbivore-induced plant volatiles in a predatory mite population. J. Chem. Ecol..

[85] Pelosi P., Zhou J.J., Ban L.P., Calvello M. (2006). Soluble proteins in insect chemical communication. Cell. Mol. Life Sci..

[86] Zhou J.J. (2010). Odorant-Binding Proteins in Insects.

[87] Olsson S.B., Linn C.E., Roelofs W.L. (2006). The chemosensory basis for behavioral divergence involved in sympatric host shifts II: Olfactory receptor neuron sensitivity and temporal firing pattern to individual key host volatiles. J. Comp. Physiol. A Neuroethol. Sens. Neural Behav. Physiol..

