# Supplementary material for: Identification and Expression Profiling of Peripheral Olfactory Genes in the Parasitoid Wasp Aphidius ervi (Hymenoptera: Braconidae) Reared on Different Aphid Hosts
Source: Insects. 2019 Nov 8;10(11):397. doi: 10.3390/insects10110397 (PMC6920860; doi:10.3390/insects10110397)
Supplement: Supplementary file 1 [file insects-10-00397-s001.pdf]

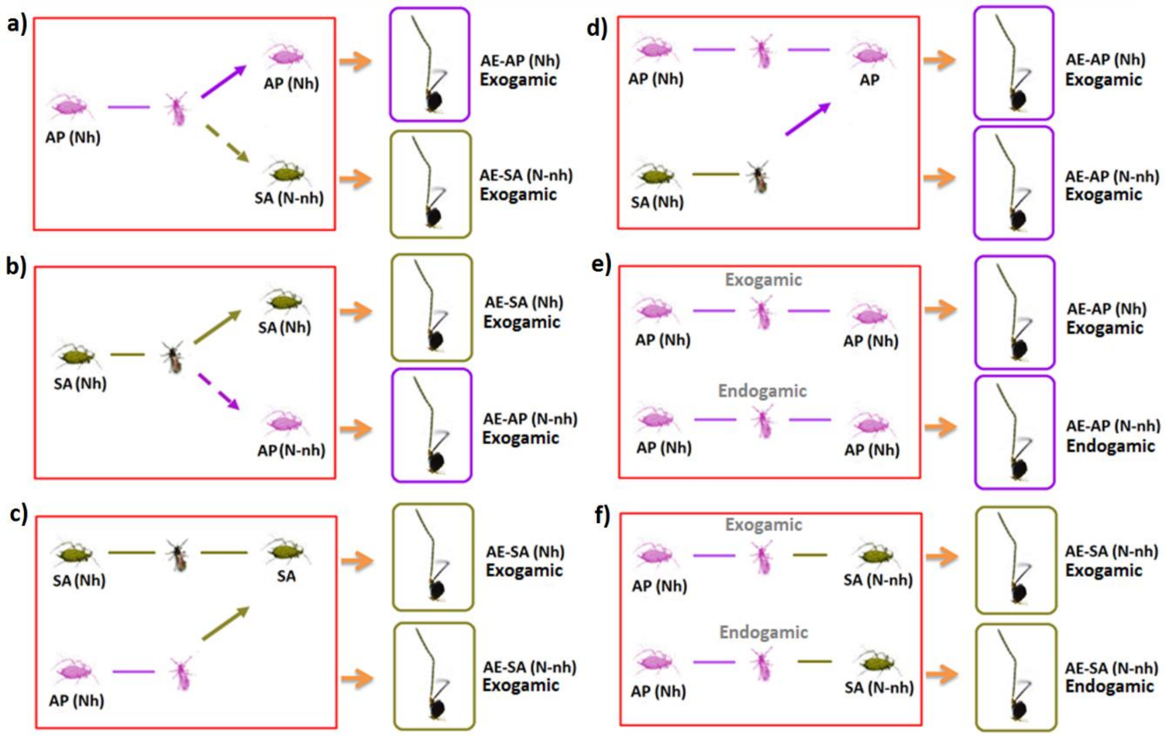

Supplementary Figure 1. Reciprocal transplant experiment of exogamic populations of *Aphidius ervi* (panels a, b, c, d) and comparison with endogamic populations on the same hosts (Sepúlveda et al. 2017a; panels e, f). **Ae:** *A. ervi;* **AP:** *A. pisum*; **SA:** *S. avenae*; **Nh**: Natal host; **N-nh**: non-natal host. **a)** Ae–AP maintained in natal host AP (Ae-AP Nh) and switched to non-natal host SA (Ae-SA N-nh). **b)** Ae-SA *S. avenae* maintained in natal host SA (Ae-SA Nh) and switched to non-natal host AP (Ae-AP N-nh). **c)** Ae-AP maintained in natal host SA (Ae-SA Nh) and Ae–SA switched to non-natal host AP (Ae-AP N-nh). **d)** Ae-AP maintained in natal host AP (Ae-AP Nh) and Ae-SA switched to non-natal host AP (Ae-AP N-nh). **e) Ae-AP** (natal host) comparison between exogamic and endogamic (75+ generations) populations. **f) Ae-SA** (non-natal host) comparison between exogamic and endogamic (75+ generations) populations.
